# Binding of Gold(III) Porphyrin by the Pro-metastatic Regulatory Protein Human Galectin-3

**DOI:** 10.3390/molecules24244561

**Published:** 2019-12-12

**Authors:** Vanya Bogoeva, Miroslav Rangelov, Nadezhda Todorova, Annie Lambert, Clarisse Bridot, Anna Yordanova, Goedele Roos, Cyrille Grandjean, Julie Bouckaert

**Affiliations:** 1 Institute of Molecular Biology “Roumen Tsanev“-Bulgarian Academy of Sciences (IMB-BAS), George Bonchev Street, bl. 21, 1113 Sofia, Bulgaria; anna_gurova@bio21.bas.bg; 2 Institute of Organic Chemistry with Center for Phytochemistry-Bulgarian Academy of Sciences (IOCCP-BAS), George Bonchev Street, bl. 21, 1113 Sofia, Bulgaria; marangelov@gmail.com; 3 Institute of Biodiversity and Ecosystem Research-Bulgarian Academy of Sciences (IBER-BAS), 2 Yuri Gagarin Street, 1113 Sofia, Bulgaria; nadeshda@abv.bg; 4 University of Nantes, CNRS, Unit for Functional and Engineered Proteins (UFIP), UMR 6286, F-44000 Nantes, France; Annie.Lambert1@univ-nantes.fr (A.L.); Cyrille.Grandjean@univ-nantes.fr (C.G.); 5 Unit for Structural et Functional Glycobiologie (UGSF), UMR 8576 of the University of Lille and CNRS, 50 Av. De Halley, 59658 Villeneuve d’Ascq, France; clarisse.bridot@univ-lille.fr (C.B.); goedele.roos@univ-lille.fr (G.R.)

**Keywords:** gold porphyrin, galectin-3, cytotoxicity, tumor associated carbohydrate antigen, affinity, microscale thermophoresis, tryptophan fluorescence, isothermal titration calorimetry, molecular dynamics relaxation, molecular docking

## Abstract

Gold(III) porphyrin presents an attractive alternative to the use of, for example, cisplatin in chemotherapy. However, approaches that allow to selectively target cancer cells are highly sought. Many plant and mammalian lectins have been shown to bind oligosaccharide sequences of the aberrant glycosylation pattern found on cancerous tumors. For example human galectin-3, of the galectin family specific for β-galactoside, is overexpressed in the extracellular matrix of tumorigenous and metastatic tissues. We searched for non-carbohydrate ligands for galectin-3 that can guide a cytotoxic drug to the cancer cells by maintaining its affinity for tumor associated carbohydrate antigens. Previous findings showed that zinc tetrasulfonatophenylporphyrin can bind galectin-3 with sub-micromolar affinity without disturbing lactose binding. Gold(III) porphyrin is not only cytotoxic to cancer cells, it knows also a potential application as photosensitiser in photodynamic therapy. We investigated the binding of gold(III) porphyrin to galectin-3 using different biophysical interaction techniques and demonstrated a low micromolar affinity of human galectin-3 for the cytotoxic compound. Co-crystallization attempts in order to understand the binding mode of gold porphyrin to galectin-3 failed, but molecular docking emphasized a highly populated secondary binding site that does not hinder lactose or Thomsen Friendenreich disaccharide binding. This suggests that gold(III) porphyrin might significantly enhance its concentration and delivery to cancer cells by binding to human galectin-3 that keeps its orientation towards tumor associated carbohydrate antigens.

## 1. Introduction

Galectins form a lectin family broadly expressed in Nature, including in mammals [1,2]. They are defined by their β-galactoside binding ability mediated by a canonical lectin domain of about 130 amino acids with a β-sandwich fold. Of note, galectins can exert their functions both extra- and intracellularly, where they play different roles in regulating protein-protein, protein-glycan and protein-membrane interactions [3]. They play a fundamental role in cell adhesion and signaling, inflammation and tumor progression and there is an enormous interest in the evaluation of galectin-glycan interactions regulating those functions [4,5,6,7,8]. 

Galectin-3 (Gal3) is unique among vertebrate galectins, because of its chimeric structure that allows it to form different types of oligomers upon ligand binding [9]. The protein consists of three parts: 1) an N-terminal 12-amino acid leader sequence containing two phosphorylation sites, 2) nine non-triple-helical collagen-like proline- and glycine-rich repeats and 3) a unique carbohydrate recognition domain (CRD) [9,10,11]. The first few amino acids forming the leader peptide are important for the subcellular localization and secretion of the protein [12]. Phosphorylation of serine in the leader peptide of human Gal3, at positions 6 and 12, plays no direct role in the affinity for its glycoprotein ligands and cellular binding of Gal3 [13].

Gal3 molecules bind to each other through the N-terminal domain, most likely into pentamers, where all carbohydrate recognition sites are available for binding glycans [9,14]. N-type self-association enables Gal3 to cluster its receptors in the plasma membrane of a cell surface [13,15]. Gal3 can also self-associate by means of its C-terminal CRD domain: in its interaction with the asialofetuin glycoprotein, the binding of a first asialofetuin-bound Gal3 molecule nucleates its association to another Gal3 molecule by means of the carbohydrate recognition site [16]. C-type self-association was recently observed in a crystal structure of a human Gal3 CRD version with inclusion of the three last (VIII-IX) Pro/Gly-rich repeats, where the last segment (IX) folded into a hairpin stabilizing Gal3 CRD tetramers in a β-sheet-like hydrogen-bonding network [17]. Intriguingly, the concave sugar-binding or S-face of the Gal3 CRD β-sandwich forms a central corridor in the tetramer, that can hold the four bound lactose molecules.

The gene encoding human Gal3 was first characterized in breast carcinoma [18]. Gal3 binds to glycoproteins and glycosylated cancer antigens on the endothelial cell surface or extracellular matrix via its carbohydrate recognition domain [11,19]. Sites adjacent to the *N*-acetyllactosamine (LacNAc) anchoring site may enhance or decrease affinity for natural β-galactoside-containing glyco- conjugates, for example repetitive LacNAc (type II)–structures (poly-LacNAc) [20,21] and the Galili antigen (Galα1,3Gal) [22] show higher affinity and specificity for Gal3 CRD compared to a single LacNAc unit. The Gal3 CRD tolerates extensions at the galactose 3′-OH-group, which can be seen for example in the co-crystal structure with α2,3-sialyllactose [21], due to its extended binding pocket. 

Gal3 plays a fundamental role in cell adhesion, inflammation and tumor progression [19] and extracellular Gal3 binds CD98 on macrophages [23,24], CD66a and CD66b on neutrophils [25] and to the T-cell receptor [23,26]. Most of those biological activities are performed by full-length Gal3, displaying the importance of glycan binding and oligomerization of the protein [27,28]. An interesting mode of action is when matrix metalloproteinases or bacterial collagenases cleave the N-terminus from Gal3, generating galectin-3C. Because galectin-3C lacks the N-terminus needed for type-N self-association and clustering of cellular receptors [13], it has no direct cellular activity. Instead, galectin-3C potentiates Gal3 binding to neutrophils using type-C self-association [29]. It thereby terminates the cellular activation by full-length Gal3, thus limiting the production of active oxygen species by neutrophils and tissue damage. 

Metal-bearing porphyrins attract scientific attention because they are cytotoxic towards cancer cells and at the same time certain complexes can be used as photosensitizers in photodynamic therapy [30,31]. For example, it has been recently reported that gold tris(carboxyphenyl) corroles have exhibited substantial phototoxicity against AY27 rat bladder cancer cells, suggesting the potential application of gold(III) porphyrines in photodynamic therapy [32]. Gal3 is an interesting target in photodynamic therapy because Gal3 overexpression is a predominant feature of many cancers. Whereas Gal3 is widely expressed in human tissues, including immune cells, epithelial cells and sensory neurons [4,25], it shows a more specific expression pattern during early stages of human embryogenesis, mainly in the epithelia, kidney, chondrocytes and the liver [11]. There is also a general shift of Gal3 localization from nucleus to the cytoplasm in cancer development from adenoma to carcinoma [26,27]. There is strong evidence showing the involvement of altered Gal3 expression and localization in the regulation of a broad range of cancer cell activates, such as tumor progression and metastasis [33], apoptosis [34], immunosuppression [35], angiogenesis [27], adhesion [15,19], invasion [36] and metabolic disorders [37].

Lectins have been well studied for their non-covalent association with porphyrins and with photosensitizers in general (for a recent review see [38]), and crystal structures of concanavalin A [39], peanut agglutininin [40] and jacalin [41] plant lectins in complex with *meso*-tetrasulfonato- phenylporphyrin (H(2)TPPS) show very convincing evidence of the attractiveness of their carbohydrate binding site for the porphyrin [39,40,41]. However, H(2)TPPS binds by intruding, overlapping or allosterically hindering the carbohydrate-binding sites of concanavalin A, peanut agglutininin and jacalin, respectively, therefore porphyrin binding might negatively interfere with the highly selective cancer-antigen targeting function of the plant lectins [38]. An earlier study demonstrated that zinc tetrasulfonatophenylporphyrin (Zn^2+^TTPS) binds Gal3 with high affinity (K_d_ = 0.18 μM) [42], largely surpassing *N*-acetyllactosamine, that is generally considered to be a specific binder of Gal3, with reported affinities K_d_ = 25 μM [43], 28 μM [44], 112 μM [45] and 118 μM [46]. Most importantly, lactose did not inhibit Zn^2+^TTPS binding [42], suggesting that carbohydrate binding might be compatible with the binding of the porphyrin photosensitizers. Nevertheless, lactose has a much lower affinity for Gal3 compared to the porphyrin, with measured equilibrium dissociation constants (K_d_) ranging from of 89.7 μM [43] to 231 μM [47]. We wanted to further investigate the molecular interactions of Gal3 with porphyrin-based photosensitizers to evaluate whether simultaneous binding of porphyrin and the tumor associated carbohydrate antigen to Gal3 would be tolerated. Aiming at this, we investigated the interactions of pro-metastatic protein Gal3 with a gold porphyrin (Au^3+^TTPS), using different biophysical techniques. Our studies revealed specific interactions between Gal3 lectins and Au^3+^TTPS, characterized by low micromolar affinities. 

## 2. Results

### 2.1. Human Galectin-3 Binds Au^3+^TTPS, but Not the Anti-Cancer Drug Roscovitine

**Figure 1:**
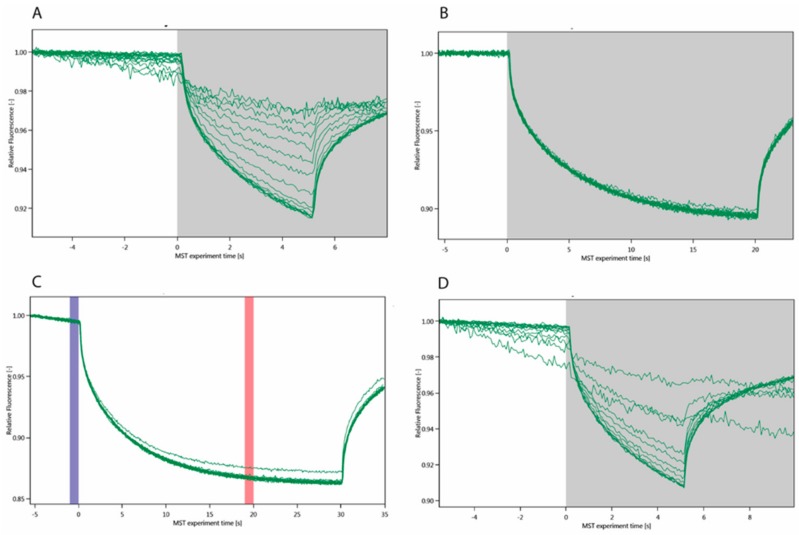
MST curves of sixteen ligand concentrations for (**A**) Gal3 FL–Au^3+^TTPS, K_D_ = 2.84 ± 1.03 μM; (**B**) Gal3 FL after SDS treament (SD test); (**C**) Gal3 FL with roscovitine: the blue band indicates the measurement of the initial fluorescence (capillary scan), before the infrared laser is illuminated to initiate the thermophoresis, the red band is the read-out of the experiment. (**D**) Gal3 CRD–Au^3+^TTPS interaction using MST.

Microscale thermophoresis (MST) is a very sensitive, immobilization-free interaction technique that is based on the speed of migration away from a central point in the capillary that is hit by an infra-red laser at time 0 (Figure 1).

The thermophoretic behavior of full-length Gal3 (Gal3 FL) (Figure 1A) and the carbohydrate recognition domain of Gal3 (Gal3 CRD) (Figure 1D) in the presence of a series of concentrations of Au^3+^TTPS were recorded, as well as Gal3 FL with roscovitine (Figure 1C). Roscovitine is a small, purine-based inhibitor of cyclin-dependent kinases that is currently being evaluated as a potential drug to treat cancers, inflammation and viral infections [48]. It was however obvious that there is no affinity between Gal3 FL and roscovitine. An initial decay in fluorescence was already observed before thermophoresis, during the capillary scan, for Gal3 FL but even more so for Gal3 CRD (Figure 1). This could potentially be caused by binding of Au^3+^TTPS too close to the fluorescent labelled lysine, or by its direct interaction with the fluorescent label (NT-647-NHS). To exclude the latter, an SD test that denatures the protein, was performed on Gal3 FL and the same series of concentrations of Au^3+^TTPS were run without observing binding (Figure 1B), indicating that a labelled lysine was likely present close to the Au^3+^TTPS binding site.

### 2.2. Truncated Galectin-3 Binds at Least 5-fold Weaker to gold(III) Porphyrin 

Although the MST curves would output an affinity, K_D_ = 2.84 ± 1.03 μM, for the Gal3 FL – Au^3+^TTPS interaction, we were cautioned by the decrease of the initial fluorescence and decided to exploit this effect of Au^3+^TTPS binding close to the labelled lysines. The fit to the plot of the capillary scan values of the initial fluorescence (time point 0 in Figure 1) for Gal3 FL and Gal3 CRD indicated that Gal3 CRD binds at least five times weaker than Gal3 FL. 

**Figure 2:**
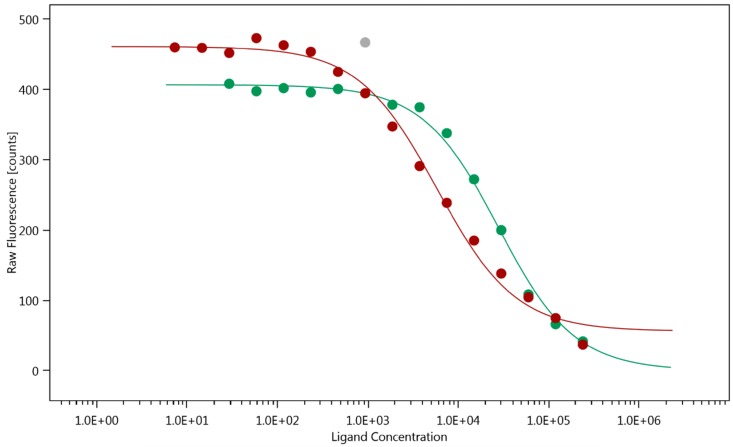
Comparison of the fit to the KD of initial (before thermophoresis) fluorescence values between Gal3 CRD (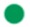) with KD = 25.90 ± 0.65 μM (outlier marked by 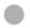) and Gal3 FL (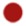) with KD = 5.83 ± 0.65 μM.

The large and unwanted initial fluorescence changes (Figure 1D) are unlikely to originate from Trp181 near the lactose-binding site of Gal3 CRD, because the MST was run using red fluorescence excitation and emission wavelengths. Also, although Gal3 FL has three tryptophans (Trp22, Trp26 and Trp181) instead of a single one (Trp181) in Gal3 CRD, it appears less affected (Figure 1A versus D). Therefore, there is more likely an involvement of NT-647-NHS-labelled lysine residues near the binding site of Au^3+^TTPS. This involvement of labelled lysines enabled the fit based on the initial fluorescence data (Figure 2). On the other hand, it would be very interesting to understand whether Au^3+^TTPS could occupy the lactose binding site of Gal3 CRD, of which its single Trp181 makes part. Therefore, we initiated tryptophan fluorescence spectroscopy measurements (TFS). 

### 2.3. Tryptophan Fluorescence Spectroscopy and Isothermal Titration Calorimetry Confirm the Low Micromolar Affinity of Au^3+^TTPS for Gal3, with a Molar Ratio of 1:1

**Figure 3:**
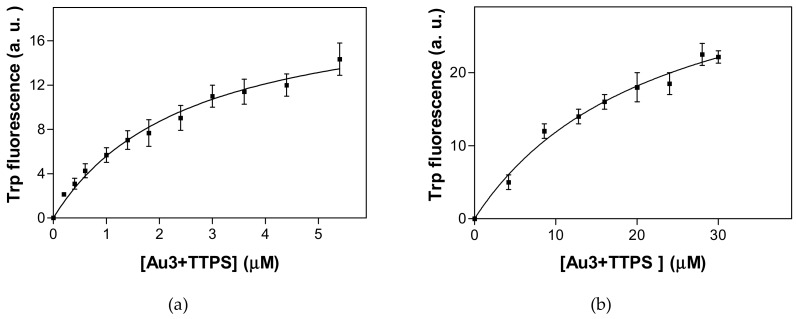
TFS measurements of complex formation with Gal3. (**a**) 2 μM Gal3 FL was titrated with 0.2–5.6 μM Au^3+^TPPS. (**b**) 10 μM Gal3 CRD was titrated with 0.2–30 μM Au^3+^TPPS. The data points were plotted as means ± standard error of the mean for three independent experimental data sets.

The formation of Gal3–Au^3+^TTPS complexes can be followed by the changes in the intrinsic or tryptophan fluorescence spectroscopy [43,49]. Binding of the metal-based drug to Gal3 FL caused a red shift of the maximum emission wavelength of about 9 nm (339 nm to 348 nm), as well as a decrease in fluorescence intensity. This is indicative of indole side chains of Trp residues being exposed on the protein surface. The interaction of the drug with Gal3 significantly influenced the fluorescence intensity: For Gal3 CRD, a decrease of fluorescence intensity was registered due to the binding, similar as for the Gal3 FL–Au^3+^TPPS complex formation, but with a smaller red shift of the emission maximum, from 341 nm to 344 nm. The fit through the data points using a non-linear regression analysis delivers a K_D_ of 2.6 ± 0.6 μM for the Gal3 FL–Au^3+^TPPS interaction and a K_D_ of 22.7 ± 5.6 μM for the Gal3 CRD–Au^3+^TPPS interaction (Figure 3). The hyperbolic nature of the binding curves of gold porphyrin to of Gal3 FL and Gal3 CRD is indicative of the presence of a single binding site for the ligand.

**Figure 4:**
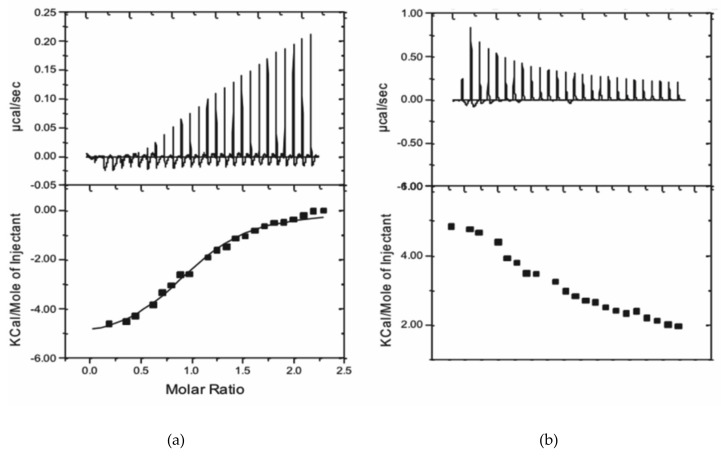
ITC experiments to measure the stoichiometry of the Gal3 FL – Au^3+^TTPS interaction and the enthalpic and entropic contributions to the binding. (**a**) The fit to the integrated heat peaks indicates a K_D_ of 3.78 ± 0.27 μM. (**b**) Subtraction of the titration with porphyrin in buffer alone was necessary because of the large, endothermic dilution heat of Au^3+^TTPS.

The number of binding sites of Au^3+^TPPS per Gal3 FL required further investigation, because higher stoichiometric ratios or porphyrin over lectin have repeatedly been observed in porphyrin stacks on the cross-links of the crystal lattice, replacing protein-protein crystal packing contacts [39,40,41]. We used isothermal titration calorimetry (ITC), which is a molecular interaction technique ideal for the reliable determination of stoichiometric ratios in the protein soluble state. The results show that both enthalpy and entropy contribute to the binding of Au^3+^TPPS to Gal3 FL (Figure 4). The fit to the integrated heat peaks data renders a stoichiometry of 1.02 ± 0.03 sites, or a single Au^3+^TTPS binding per Gal3 FL and an equilibrium dissociation constant K_D_ of 3.78 ± 0.27 μM at 22 °C, corresponding to a change in Gibbs free energy (ΔG) of −7326 cal/mol. The reaction is exothermic and enthalpy driven with a heat release of −5363 ± 196.4 cal/mol, indicating a substantial number of non-covalent bonding during complex formation. Moreover, the interaction also profits from a significant entropic contribution of 1956 cal/mol.

### 2.4. Molecular Dynamics Relaxation of the Gal3 CRD Structure and Docking of Au^3+^TPPS 

**Figure 5:**
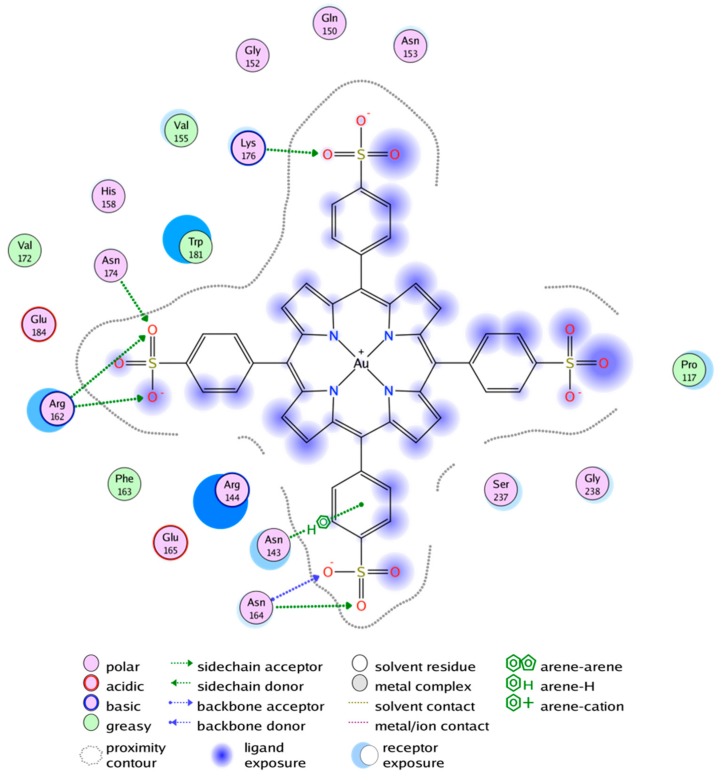
The nature of the interactions of the best scoring Au^3+^TPPS docking pose with Gal3 CRD is as described in the legend. This first docking site coincides with the lactose-binding site of Gal3 CRD.

In silico results were not significantly different between the two chosen Gal3 CRD conformations and demonstrated only two potential docking sites for Au^3+^TPPS, both presented on the concave sugar-binding S-side of the Gal3 CRD β-sandwhich (Figure 5). The majority of the interactions are electrostatic due to the four negatively charged sulfonatophenyl groups of ion-TPPS molecules and an abundance of arginine and lysine side chains on the S-face of the Gal3 CRD. 

The ranking of docking poses are listed values from the GBVI/WSA scoring function as described [50] and is expressed in ΔG [cal/mol]. Two sites on the S-face of Gal3 CRD that can bind metal ion-porphyrin complexes are identified by this docking procedure.

**Figure 6:**
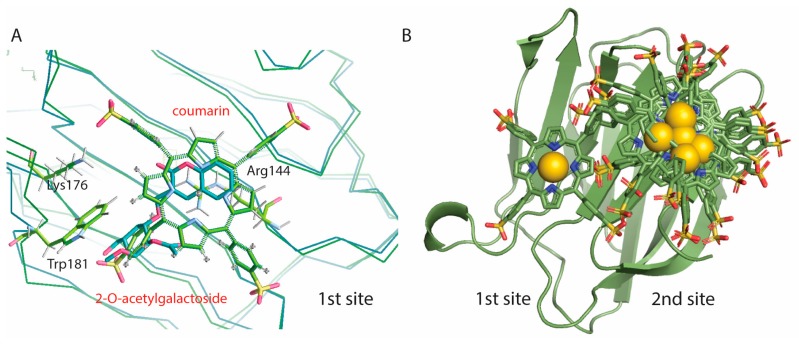
Gold(III) porphyrin (Au^3+^TTPS) docks in two sites on Gal3 CRD. (**A**) Only the two highest energy poses of Au^3+^TTPS cover the lactose-binding site of Gal3 CRD, where the porphyrin ring stacks on top of Arg144 similar to the coumarin moiety of the coumaryl 2-*O*-acetylated galactoside Gal3 inhibitor (PDB entry 5exo [51]). (**B**) In the other eight poses, Au^3+^TTPS stacks on top of the Asn119 amide, remote from the lactose-binding site. The Au^3+^ ion is represented as a golden sphere.

The first two poses that have the most favorable binding energies, of −7971 cal/mol and −7678 cal/mol respectively, superimpose onto the carbohydrate binding site, which we call docking 1st site (Figure 6). Binding of the porphyrin here would block the galectin’s ability to bind carbohydrates (Figure 6A). The next three poses have slightly lower docking energies, from −7436 cal/mol to −6954 cal/mol, and Au^3+^TPP is docking on a second site of the S-face, on a position that is not involved in carbohydrate analog binding (Figure 6B). The next five poses with even lower energy dock in this same 2nd site.

Figure 6B illustrates that for small carbohydrates, for example LacNAc or the Thomsen Friedenreich (TF) disaccharide, carbohydrate and Au^3+^TPPS binding can probably function simultaneously by using the 2nd site for the porphyrin.

## 3. Discussion

Medicinal applications of metal complexes as therapeutic drugs have a long history, dating back to over 5,000 years ago. The development of gold(III) complexes as potential anti-cancer drug with higher cytotoxicity on cancer cells than existing metal anticancer drugs has been actively studied in recent years. Several gold(III) complexes have been reported to exhibit cytotoxicities against tumor cells, and are at least comparable with cytotoxicity of cisplatin [52]. Moreover, porphyrins do not cause severe side effects, which makes them more effective than other chemotherapeutic drugs. Gold(III) porphyrin was effective in killing multidrug-resistant KB-V1 human oral epidermoid carcinoma cells as well as cisplatin-resistant CNE-1 human nasopharyngeal carcinoma cells [53]. It induced apoptosis and prolonged the survival of hepatocellular carcinoma-bearing rats as well as inhibited the tumor growth of mice bearing nasopharyngeal carcinoma, neuroblastoma and colon carcinoma [54]. Also it is found that gold(III) porphyrins induce extensive apoptosis in HeLa cancer cells [55]. In 2017, Dandash reported on the in vitro anticancer activity of new gold(III) porphyrin complexes in colon cancer cells [56]. These studies showed the potential of gold(III) porphyrin to act as tumor targeting reagent, which motivated the present study.

In our measurements, the affinity of the human Gal3 FL for Au^3+^TPPS is consistently within the micromolar range (dissociation constant K_D_ = 2.84 μM using MST, 2.6 μM using TFS and 3.78 μM using ITC) and reveals a binding of the protein for this compound several orders of magnitude stronger than that of cisplatin for human serum albumin. The binding affinities obtained using fluorescence or calorimetry detection are therefore in good agreement, which is congruent with earlier findings for the combination of these interaction techniques for galectins [43]. Moreover, the Gibbs free energy (ΔG), directly measured using ITC, of −7326 cal/mol is close to the median binding energy of the five best poses (−7436 cal/mol) in molecular docking, distributed over the 1st/2nd site in a 2/3 ratio (Figure 6). 

The two best ranking docking poses are binding at van der Waals distance from Trp181 in the 1^st^ site, giving rise to measurable instrinsic or Trp fluorescence spectra (TFS). The two sites overlap, which is consistent with the single Au^3+^TPPS binding per Gal3 FL, obtained from the TFS curvature and the ITC molar ratio. In the TFS experiments, the red shift for the emission maximum observed upon binding Au^3+^TPPS to Gal3 FL (9 nm) is much smaller for Gal3 CRD (3 nm). This indicates that the single Trp181 in Gal3 CRD carbohydrate binding site (1st site, Figure 5) is most likely involved in the binding, but that it is not the sole reason for the fluorescence changes. Thereby it leaves the possibility of alternative binding positions for Au^3+^TTPS onto Gal3 CRD, such as the 2nd docking site (Figure 6B). Basic amino acids from the Gal-3 CRD docking sites, that are arranged in electrostatic interactions with the four sulfonatophenyl groups in ion-TPPS molecules, are Arg162, Lys176 and Lys139 for the 1st site, and Lys233, Arg144, Arg162, Lys176 for the 2nd site (Figure 5). Several interaction residues are shared between the two docking sites: Arg144 that stacks with its guanidinium group underneath the porphyrin ring in the 1st site (Figure 6A) makes an electrostatic interaction with a sulfonatophenyl when Au^3+^TPPS is bound in the 2nd site. Arg162 is in the middle between the two docking sites. Lys176 may give a large contribution to initial fluorescence decrease in the MST curvature (Figure 1), when fluorescently labelled and interacting with Au^3+^TPPS, in the 1st site (Figure 5; Figure 6A). A similar role can be played by Lys233 in the 2nd docking site.

Several conclusions on the binding characteristics of Au^3+^TPPS to Gal3 FL can be drawn from these studies. We show that both fluorescent labelling and intrinsic Trp fluorescence can serve as reliable ways to determine Gal3-ligand interactions. The measurements of binding constants obtained using MST, TFS and ITC and even molecular docking are consistent with the Au^3+^TPPS–Gal3 FL interaction of low micromolar affinity. The affinity of Au^3+^TPPS for Gal3 FL is higher than for LacNAc with one binding site but lower than for asialofetuin (K_D_ = 720 nM) with nine LacNAc epitopes [57] and is the same as for blood group type II [45]. Similar to the interaction with oligosaccharides and the asialofetuin glycoprotein, the binding of Au^3+^TPPS is enthalpically driven, however the entropic contribution to binding is also positive. One possibility is that this is due to the uncompensated charge of Au^3+^ that leads to positively charged porphyrin skeleton, in contrast to Zn^2+^TPPS [42,49] where the porphyrin skeleton is almost neutral. However, thermodynamic studies showed that the binding several metal-ion porphyrins to *Trichosanthes cucumerina* lectin TCSL was also mainly driven by the change in entropy, while the enthalpic contribution was very small [58]. Positive entropy contributions were also observed for metal-ion porphyrin binding to the jacalin (*Artocarpus integrifolia*) lectin [59].

No co-crystals of Gal3 with Au^3+^TPPS could be obtained, therefore we turned towards docking to understand the molecular recognition. We found two sites on the sugar-binding face (S-face) of the Gal3 CRD that can bind relatively well porphyrin complexes. The 1st site, slightly preferred for complexation, would block Gal33’s ability to bind carbohydrates, although tighter-binding natural glycoconjugate ligands could most likely displace it. For example, Gal3 can bind with sub-micromolar affinity to physiological cell membrane glycoproteins as well as exogenous receptors. A very strong affinity was observed for Gal3 binding of an antifreeze glycoprotein from Atlantic cod (*Gadus morhua*), named Thomsen Friedenreich (TF) disaccharide-100 because of its glycosylation with the Galβ1,3GalNAcα1-Ser/Thr tumor associated carbohydrate antigen and its presumed molecular weight of 100 kDa [44]. Gal3 also recognizes the CD146, or cluster of differentiation 146, also known as the melanoma cell adhesion molecule (MCAM), cell surface glycoprotein MUC18 (mucin-18) or Melanoma cell adhesion molecule, Mel-CAM, found in smooth muscle, vascular endothelium, cerebellum, hair follicles, activated T-cells and bone marrow, with sub-micromolar affinity (K_D_ = 0.43 μM, measured using bio-layer interferometry). Similar to in our ITC study, this affinity is reduced to unmeasurable values for Gal3 CRD [46]. NMR data moreover revealed that, although the canonical sugar-binding β-sheet, or S-face, of Gal3 is involved in the interactions with CD146, the other β-sheet or F-face of Gal3 also undergoes significant spectral perturbations when binding CD146 [46]. The direct role for the galectin’s CRD F-face in the CD146 binding process was further corroborated by the finding that the addition of lactose, known to bind the S-face, significantly attenuated Gal3 binding to CD146, but could not abolish it. This is reminiscent of the earlier findings that galactose and noticeably lactose are not necessarily incompatible with the binding of metal-ion porphyrins to lectins [42,58,59]. Potentially, porphyrin ligands play a role in self-association of Gal3 in analogy with galectin-3C [29].

Gal3 plays an important role in fibrotic diseases, eg. cardiac fibrosis, which is in the basis of the development of heart failure, and pulmonary fibrosis [60,61]. Coumaryl 2-*O*-acetylated galactoside, that targets the Gal3 glycan recognition, can interfere with the Gal3-promoted sustained profibrotic cell signaling and scar formation [51] and we show that Au^3+^TPPS can bind this same site (Figure 6A). Here, Au^3+^TPPS has the potential as a Gal3 inhibitor or as well as a photosensitizer in photodynamic therapy [38]. The 2nd docking site for Au^3+^TPPS is placed on a part of the Gal3 molecule that is not known to be directly involved in carbohydrate binding (Figure 6B). Small carbohydrates, such as the generally short tumor associated carbohydrate antigens, and the metal ion porphyrin can in principle bind simultaneously. Au^3+^TPPS could use Gal3 as a vehicle to guide it towards cancer cells where it can exert its cytotoxicity. The obtained results have shown that Gal3 interacts with gold(III) porphyrin with high affinity.

In conclusion, the present study characterizes the capacity of the human Gal3 lectin to bind non-classical anti-cancer compounds [42], in particular Au^3+^TPPS that appears to be an appropriate alternative to classical anti-cancer compounds [52,53,54,55,56], and reveals new perspectives in the drug delivery of anti-cancer agents [62].

## 4. Materials and Methods

### 4.1. Materials

Recombinant human galectin-3 (Gal3, 26 kDa, full-length galectin-3 or Gal3 FL) was prepared as described previously [6]. The carbohydrate recognition domain (Pro106-Ile250) of human Gal3 (Gal3 CRD), was cloned in the pET15 vector and expressed in *Escherichia coli* BL21(DE3) following an exponential phase induction with 100 µM of isopropyl β-d-1-thiogalactopyranoside (IPTG) overnight at 20 °C. The Gal3 CRD is purified in 50 mM Hepes pH 7.5, 100 mM NaCl, 2 mM DTT, using His-tag affinity chromatography on Protino Ni-NTA agarose (Macherey-Nagel, Dueren, Germany), followed by size exclusion chromatography on a HiLoad 15/600 Superdex^TM^ 75pg resin (GE Healthcare, Wauwatosa, WI, USA) in PBS buffer at pH 7.4 [4].

The adenine derivative roscovitine (CAS Number 186692-46-6) and all chemicals, if not otherwise mentioned, were purchased from Sigma-Aldrich (Saint Louis, MO, USA) and its concentration in 10% ethanol was determined by measuring ultraviolet absorption at 255 nm and conversion using the molar extinction coefficient, ε_M_, 255 nm = 7660 M^−1^ cm^−1^. 5,10,15,20-Tetrakis(4-sulfonatophenyl)-porphyrin-Au(III)chloride (Au^3+^TPPS) was obtained from Porphyrin Systems (Lüebeck, Germany). The concentration of gold porphyrin was calculated by measuring absorbance at 403 nm and applying the conversion ε_M_, 403 nm = 2.82 × 10^5^ M^−1^ cm^−1^.

### 4.2. Methods

#### 4.2.1. Microscale Thermophoresis

Microscale thermophoresis (MST) is a technique to quantify biomolecular interactions by measuring the directed movement of molecules in a microscopic temperature gradient induced by an infrared laser. The directed movement of molecules in thin capillaries was detected and quantified by means of a red-light fluorophore (NT-647-NHS, with excitation maximum at 647 nm and emission maximum at 670 nm) covalently linked to Gal3 proteins. For labelling, we used two times the concentration of the protein (16 μM Gal3 FL, 43 μM Gal3 CRD) for the fluorophore. Removal of excess fluorphore was combined with buffer exchange into 20 mM Hepes, pH 7.4 with 150 mM NaCl and 0.05% Tween-20.

A two-fold dilution series of ligand concentrations was established ranging from 239 μM to 7.3 μM, with a constant concentration of 4 μM Gal3 FL and 22 μM Gal3 CRD. The samples were loaded into standard glass capillaries and readings were performed with the red light of a Monolith NT.115, with LED power setting of 20% and an MST power setting of 40%. Experiments without and with prior sodium dodecyl sulfate (SDS) denaturation (4% SDS, 40 mM dithiothreitol (DTT), with boiling at 95 °C) have been performed twice for the Au^3+^TPPS -Gal3 FL interaction. The same procedure was repeated for Gal3 CRD and using roscovitine as a ligand. The data were analyzed using the MO Affinity Analysis software v2.2.4.

#### 4.2.2. Intrinsic or Tryptophan Fluorescence Spectroscopy

Tryptophan fluorescence spectroscopy (TFS) was performed on a Shimadzu spectrofluorometer (Shimadzu, Kyoto, Japan). In order to avoid detection of tyrosine emission, the protein samples were excited at 295 nm with an excitation band pass of 5 nm and an emission band pass of 10 nm. Total fluorescence was calculated after normalization of the fluorescence spectra and corrected for dilution. In order to account for the inner filter and the self-absorption effects, the experiments were always carried out on samples with absorbance (OD_280nm_) less than 0.05. Absorbance was measured using a spectrophotometer (Beckman, Brea, CA, USA). All measurements were performed at 25 °C, with the temperature of the samples determined in the cuvette with an accuracy of ± 0.2 °C. Drug-Gal3 interactions were measured by titrating increasing concentrations (0.2–5.6 μM) of Au^3+^TPPS into 2 μM Gal3 FL and 10 μM Gal3 CRD, using a 20 mM phosphate buffer containing 0.15 M NaCl, pH 7.4) and the data were analyzed using non-linear regression with the PRISM software.

#### 4.2.3. Isothermal Titration Calorimetry

A VP-ITC MicroCal instrument (Malvern Panalytical Ltd, Malvern, UK) with cell volume of 1.4253 mL was used at 22 °C for the measurement of the stoichiometric ratio and the enthalpy/entropy contributions to the binding of gold prophyrin to Gal3 FL. Prior to the experiment, the Gal3 FL protein was dialyzed extensively against 20 mM Hepes at pH 7.4 and 150 mM NaCl, the buffer used for the experiment. Au^3+^TPPS (389.34 μM) was titrated in 10 μl aliquots into Gal3 FL or Gal3 CRD protein solutions (32.67 μM). A separate titration of 10 μL aliquots of Au^3+^TPPS (389.34 μM) solution into buffer, using the same time intervals (400 s) was used to measure the dilution heat peaks from Au^3+^TPPS alone, and these were subtracted from the interaction peaks. Titration of gold(III) prophyrin to Gal3 CRD gave no measurable results, also not upon subtraction of the heat peaks from Au^3+^TPPS dilution into buffer. Twice higher concentrations of Gal3 CRD and titrant did not solve this issue, likely because the dilution heat peaks from Au^3+^TPPS were too dominant and cancelled out the heat released from the weak binding reaction.

#### 4.2.4. Co-Crystallization Trials of Gal3 with Gold(III) Porphyrin

Crystalllization trials by co-incubation of 1.2 mM Au^3+^TPPS with either Gal3 FL or Gal3 CRD at 12 mg/mL concentration were set up, using the sitting drop and hanging drop vapor diffusion technique with a 1:1 volume ratio protein-ligand:precipitant solutions. The precipitants in the PACT Premium, MIDAS, JCSG-Plus, Memgold and PGA 96-well high-throughput crystallization screens (Molecular Dimensions, Newmarket, UK) have been tested. Only Au^3+^TPPS crystals could be obtained, in hanging drops containing 1 μL of the protein-ligand mixture and 1 μL of 15% *w/v* polyacrylate sodium salt 2100, 0.2 M magnesium chloride, 0.1 M Hepes-NaOH at pH 7.5. This condition led to crystals that did not light up under ultraviolet light (UV Bench, NatX-ray, Saint Martin d′Hères, France) and that also did no diffract X-rays like protein crystals. Their hexagonal shape and the X-ray diffraction characteristics strongly suggests that these crystals are liquid crystals of stacked gold(III) porphyrin molecules, as recently described [63].

#### 4.2.5. Molecular Dynamics Relaxations of Gal3 CRD and Docking of Porphyrins

In order to obtain Gal3 structures for the further docking of porphyrin molecules, we performed a molecular dynamics (MD) simulation on an initially corrected and electroneutral Gal3 CRD (PDB entry code 5exo, [51]). The MD relaxation was run using the GROMACS 5.0 software package and the CHARMM27 force field with explicit TIP3P (transferable intermolecular potential with 3 points) water molecules and with a physiological salt concentration of 154 mM NaCl. After equilibration of the system at 300K and 1 atm in an NpT (isobaric-isothermic) ensemble, the final 30 ns of the trajectory was subject to cluster analysis and the two central Gal3 CRD conformations of the two major clusters from that trajectory were input for the molecular docking simulations. 

In the Au^3+^TPPS model used for docking, the distance between two opposite nitrogen atoms is 4.137 Å for the Au^3+^ porphyrins and the distance between two opposite carbon atoms carrying the sulphonatophenyl group is 6.996 Å. We compared our structures with structures generated by Density Functional Theory (DFT) calculations and we found that the difference between DFT calculations and our structures is due to the used AMBER12 force field and the Extended Hückel theory (EHT) used for Au^3+^TPPS molecular structure generation and in post-docking Induced Fit calculations. Geometric restraints were included in order to keep the Au^3+^TPPS chelate flat as was predicted by Density Functional Theory DFT calculations. The docking simulations were performed using the MOE 2016 software. Primary placement was performed by Alpha triangle algorithm and the selected best poses were further processed using an Induced Fit algorithm. The GBVI/WSA dG (Generalized-Born Volume Integral/Weighted Surface area) [nm] scoring function was used for scoring the energetic likeliness of the poses [50].
